# Vibrotactile sensitivity of patients with HIV‐related sensory neuropathy: An exploratory study

**DOI:** 10.1002/brb3.1184

**Published:** 2018-12-18

**Authors:** David Karpul, Sarah McIntyre, André van Schaik, Paul P. Breen, Jeannine M. Heckmann

**Affiliations:** ^1^ MARCS Institute for Brain, Behaviour & Development Western Sydney University Sydney New South Wales Australia; ^2^ Division of Neurology, Department of Medicine University of Cape Town Cape Town South Africa; ^3^ Neuroscience Research Australia Sydney New South Wales Australia; ^4^ Department of Clinical and Experimental Medicine Linköping University Linköping Sweden; ^5^ Translational Health Research Institute Western Sydney University Sydney New South Wales Australia

**Keywords:** HIV, peripheral sensory neuropathy, QST, vibrotactile sensitivity

## Abstract

**Background:**

HIV‐associated distal polyneuropathy (HIV‐PN) affects large and small sensory nerve fibers and can cause tactile insensitivity. This exploratory study forms part of an effort to apply subsensory electrical nerve stimulation (SENS) to improve tactile sensitivity of patients with HIV‐PN. This work presented an opportunity to use a robust protocol to quantitatively describe the vibrotactile sensitivity of individuals with HIV‐PN on effective antiretroviral therapy (ART) and correlate these findings with commonly used clinical vibration testing and scoring grades.

**Methods:**

The vibration perception thresholds (VPTs) of 20 patients with HIV‐PN at three vibration frequencies (25, 50, and 128 Hz) were measured. We compare the vibration perception threshold (VPT) outcomes to an age‐ and gender‐matched control cohort. We further correlated VPT findings with 128 Hz tuning fork (TF) assessments performed on the HIV‐PN participants, accrued as part of a larger study. HIV‐PN was defined as having at least one distal symmetrical neuropathic sign, although 18 of 20 had at least two neuropathic signs.

**Conclusions:**

HIV‐PN participants were found to have lower VPT sensitivity than controls for all three vibration frequencies, and VPT was more sensitive at higher vibration frequencies for both HIV‐PN and controls. VPT sensitivity was reduced with older age. Years on ART was correlated with VPT‐25 Hz but not with VPT in general. Notably, VPT sensitivity did not correlate with the clinically used 128 Hz TF severity grades. Outcomes of tests for interaction with vibration frequency suggest that HIV‐PN pathology does not affect all mechanoreceptors similarly.

## 1 INTRODUCTION

Peripheral neuropathic desensitization (PND) is a common problem that can be caused by HIV, diabetes, alcoholism, aging, and many other conditions. PND results in secondary conditions as a result of the reduced sensation. For example, plantar sensitivity affects balance (Gravelle et al., 2002; Menz, Morris, & Lord, 2005) and the risk of falling (MacGilchrist et al., 2010) which is associated with substantial morbidity in individuals over the age of 65 (Collins et al., 2003; Hoyert, Arias, Smith, Murphy, & Kochanek, 2001; Priplata, Niemi, Harry, Lipsitz, & Collins, 2003).

More than 5 million people live with HIV in South Africa (Anon, 2012), up to 60% of which suffer from HIV‐related peripheral sensory neuropathy (HIV‐PN), which may arise as a consequence of the infection or following antiretroviral therapy (ART) initiation (Centner, Bateman, & Heckmann, 2013; Centner et al., 2018). While there is still no cure for HIV, ART has significantly reduced HIV‐associated mortality. In addition, HIV‐related neurological conditions, and specifically HIV‐PN, which is more frequently painless (Centner et al., 2018), have been shown to adversely affect health‐related quality of life (Pandya, Krentz, Gill, & Power, 2005). There is a growing need to find interventions to help this group manage the consequences of HIV‐PN.

Over the last 20 years, a family of exploratory intervention studies, typically with samples of 8–16 participants, have sought to improve tactile sensitivity in healthy and neuropathic populations by applying continuous electrical or mechanical stimulation delivered at amplitudes below the perceptual threshold (Breen et al., 2016; Collins, Imhoff, & Grigg, 1997 *;* Iliopoulos, Nierhaus, & Villringer, 2014; Priplata et al., 2006 *; *and related references). These studies, however, have never examined patients with HIV‐PN.

This work forms the initial part of an intervention study where we will apply subsensory electrical nerve stimulation or SENS (see Breen et al., 2011; Breen & Macefield, 2013; Breen et al., 2014; Breen et al., 2016; Serrador, 2012) to the recruited HIV‐PN and matched control participants. The intervention study presented a unique opportunity to report on baseline levels of vibration perception, in mechanical units (microns) at different vibration frequencies, and correlate them to clinical measures already performed on the HIV‐PN cohort as part of a larger HIV “inflamm‐aging” neuropathy study (Borkum et al., 2017).

Given that this study aims to detect subclinical changes in perception thresholds, the measurements used require higher resolution than clinical tools. Before conducting the intervention, we wish to quantify the tactile sensitivity of patients with HIV‐PN using this protocol and compare the results to healthy matched controls. This in turn can be used to justify the intervention with this population.

Here, we report a detailed description of the vibrotactile sensitivity of individuals with HIV‐PN at different vibration frequencies and compare the commonly used clinical vibration testing and scoring grades with a more robust double‐blinded quantitative vibration perception threshold (VPT) protocol.

## 2 METHODS AND MATERIALS

### 2.1 Participants

Twenty two HIV‐PN participants were recruited consecutively from a cohort of a larger HIV “inflamm‐aging” neuropathy study (Borkum et al., 2017), which monitored patients after enrollment on a government‐sponsored ART program. These patients were recruited from the HIV clinic at Crossroads Community Health Centre in Cape Town, South Africa. A total of 21 healthy control participants were also recruited from the community. All participants were required to sign informed consent, which outlined the experimental protocol and exclusion criteria, provided in either English or Xhosa. This study was approved by the Human Research Ethics Committees of both the University of Cape Town (HREC ref: 838/2015) and Western Sydney University (ref: H11381).

The focused neuropathy examination was performed on the HIV‐PN group by a neurologist (JMH). The HIV‐PN group consisted of individuals with one or more neuropathic signs present in a symmetrical distribution. Patients with severe painful neuropathy scoring greater than 6 out of 10 on a visual numeric scale were excluded from the current study. Those with severe painful neuropathy were excluded for two reasons: because pain may confound the measurement of VPT, and because the hypothesis is that SENS, which aims to improve tactile sensitivity, may also increase perceived pain in these patients. The effects of SENS on neuropathic pain are currently unknown.

Control participants were age‐ and gender‐matched as a group, self‐reported as HIV negative, and had a similar demographic background, being recruited from the same geographic region.

Both groups were required to be physically able to perform the tasks in the study, be between 18 and 59 years of age, not be pregnant, have no history of cardiovascular disease or epilepsy, have no implants including pacemakers, not suffer from diabetes or alcoholism, and not be diagnosed with any illness that affects peripheral sensation aside from HIV. Control participants did not undergo neurological examination.

Participants younger than 60 years were included as sub‐Saharan African HIV cohorts are relatively young and older age may impact independently on VPT (Mold, Vesely, Keyl, Schenk, & Roberts, 2004). Participants’ height, weight, date of birth, foot dominance, and self‐reported sex were recorded. Foot dominance is the foot (left or right) most likely to be used for dexterous tasks. This was measured to arbitrarily standardize which foot was used for VPT measurements.

### 2.2 Clinical neuropathy screening

As part of the HIV “inflamm‐aging” study, the HIV‐infected participants underwent a full neuropathy screening examination. The Brief Peripheral Neuropathy Screening tool (BPNS) and a reduced version of the Total Neuropathy Screen (TNSr) were used to assess HIV patients for the presence of symmetrical neuropathic symptoms and signs both qualitatively and quantitatively (Centner et al., 2018; Cherry, Wesselingh, Lal, & McArthur, 2005; Maritz et al., 2010). Symptoms are defined as features of a disease that are apparent to the patient, and signs as features detected through medical examination.

The BPNS establishes symptoms of pain, numbness, and “pins and needles” on a visual numeric scale. It further examines vibration perception in the toes and the presence of ankle reflexes. The ankle reflexes are scored as either normal (2), reduced (3), or absent (4). Vibration perception was assessed using a standard technique of applying the maximally vibrating 128 Hz tuning fork (TF) immediately to the distal interphalangeal joint at the hallux (Cherry et al., 2005). The final result is given as an ordinal variable indicating the duration for which vibration was detectable: score 0 for >10 s, score 1 for 6–10 s, score 2 for 1–5 s, and score 3 for no detectable vibration, denoting severe loss of vibration perception.

The TNSr assesses the extent to which neuropathic symptoms and signs progress distally (toes/soles of feet (1)) to proximally (ankles (2), knees (3), hands (4)), with more proximal abnormalities scoring higher (worse). Five categories are examined: sensory symptoms, pin sensibility, vibration sensibility, deep tendon reflexes, and strength of plantar and ankle/toe dorsiflexion. In addition, proprioception was assessed at the toes, and >20% mistakes (of 10 trials) bilaterally was categorized as abnormal (Centner et al., 2018).

For the purposes of this study, we included patients consecutively, who satisfied the most widely used definition of HIV‐PN, namely the presence of at least 1 distal symmetrical neuropathic sign (see Centner et al., 2013 *; *Maritz et al., 2010 *; *Phillips et al., 2014). The parameters selected from the HIV “inflamm‐aging” neuropathy study (Borkum et al., 2017) for comparison with the VPT here were as follows: date since initiating ART, ART regimen, toe proprioception (see Centner et al., 2018), BPNS tuning fork test (BPNS‐TF), BPNS evaluation of ankle deep tendon reflexes (BPNS‐DTR), and the total BPNS score (BPNS‐total). Further, VPT was compared with the following outcomes from the TNSr: the distal–proximal extent of reduced abnormal vibration sensibility (TNSr‐TF), pinprick abnormalities, reduction in deep tendon reflexes (TNSr‐DTR), and the total TNSr score (TNSr‐total).

### 2.3 Measurement of VPT

**Figure 1:**
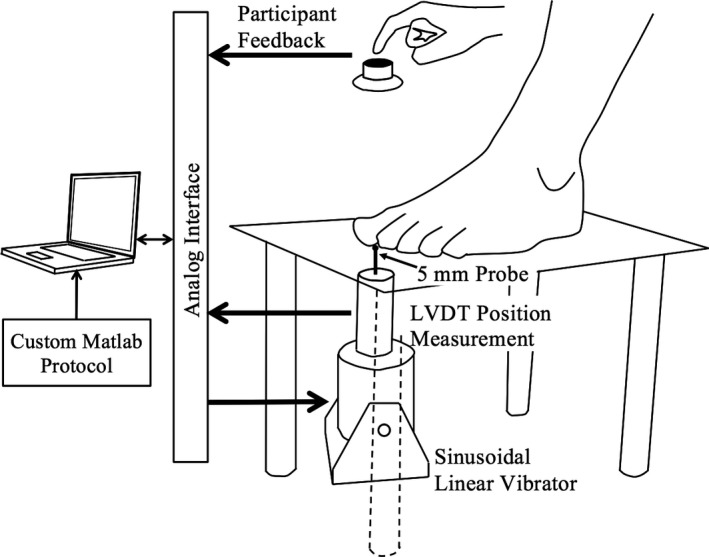
Diagram showing the testing platform and control and instrumentation setup of the vibration sensitivity testing. The platform had a 20 mm hole in its surface through which the probe made contact with the skin

Participants were asked to sit in a chair and place their nondominant foot on a platform shown in Figure 1. The underside of the big toe was placed over a 20 mm hole in the platform that allowed it to make contact with the 5 mm ball bearing‐tipped probe underneath.

A hook‐and‐loop strap over the toes was used to keep the foot in place. It also ensured that the hallux was in contact with the probe, depressing the probe by 50–150 μm. Adjustments were made during the experiment, if needed, to ensure that contact was maintained in this range.

**Figure 2:**
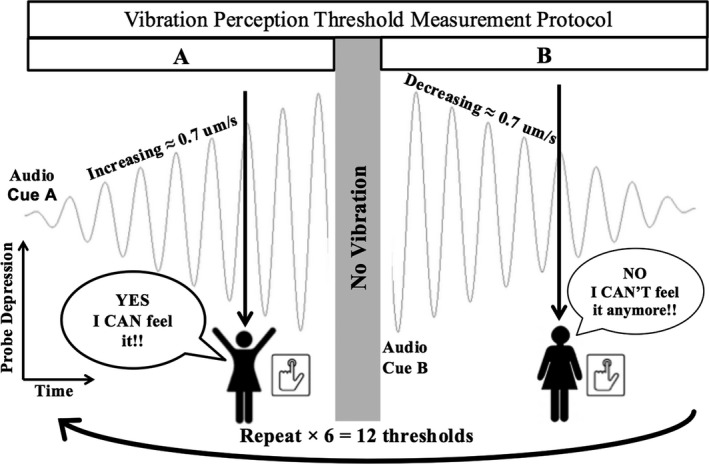
Diagram depicting the testing protocol for vibrotactile threshold testing

Figure 2 shows the modified method of limits protocol, which was conducted almost entirely by a custom written Matlab program to ensure double blinding.

Participants would first hear an easily recognizable audio cue indicating that an “up going” vibration ramp was imminent. The vibration would then start at an imperceptible amplitude and increase until the participant could feel the vibration. The participant was instructed to press a handheld button immediately upon becoming aware of the vibration. The vibration would then pause for one second, and a different audio cue would sound, indicating the “down going” vibration ramp was imminent. The vibration would then start from a suprathreshold amplitude and decrease toward zero at the same rate as before. The participant would then press the handheld button as soon as they could no longer feel the vibration. The sequence repeats until a total of 12 thresholds were collected. This would typically take between 5 and 10 min depending on the participant's thresholds and the rate of the ramps. The average vibration amplitude in the 0.5 s leading up to the button press was deemed the threshold for that ramp.

The protocol was repeated for three different vibration frequencies. These frequencies were specifically chosen to selectively activate predominantly low‐frequency mechanoreceptors (25 Hz, hereinafter referred to as VPT‐25), predominantly high‐frequency mechanoreceptors (128 Hz or VPT‐128), and combination of the two (50 Hz or VPT‐50). Further, VPT‐128 allowed for comparison to traditional tuning fork measurements.

To avoid confusion between the term “frequency” referring to occurrence rate in participants and “frequency” referring to vibration cycles per second, all references to the latter will be replaced with “Hz.”.

### 2.4 Data analysis

The comparison of the HIV‐PN and the control cohorts was conducted with a two‐sample *t* test for each continuous variable (calculated in Microsoft Excel 2016), and the Fisher's exact test comparing foot dominance (calculated in Matlab 2017a). The effects of various parameters on VPT (all three Hz) were computed as odds ratios of linear mixed effects models (calculated in “R” 3.3.1). Statistics are noted as probability (*p*), chi‐squared value (*χ* ^2^), and degrees of freedom (*df*). When testing the correlation between only two variables, such as between a single VPT Hz and a tuning fork test outcome, Spearman rank order tests were used (calculated in Matlab 2017a).

The number of participants selected for this work powered the study to find differences in VPT in a SENS intervention study to follow. The statistical power calculations were based on data presented in a similar study presented by Breen et al., 2011. A sample size of 20 in each group powers this work to find a statistically significant difference of 0.93 standard deviations between groups and regression correlations of at least 0.43 in magnitude between two continuous variables such as age and VPT.

Results are regarded as significant if the *p*‐value is <0.05. However, results below 0.15 will also be discussed in context and may justify further research.

## 3 RESULTS

### 3.1 Group characteristics

Two of the 22 HIV‐PN participants recruited were excluded from the results analysis. One was not able to learn the task, and the other had vibratory thresholds in excess of what the equipment was able to measure (>1,000 μm); she had severe HIV‐PN with absent deep tendon reflexes, unable to perceive vibration sense with the TF at the toes only, altered proprioception at the toes, and loss of pin sensibility extending to the knees. One of the 21 control participants recruited was excluded as their VPTs at different Hz were between 4.3 and 12 standard deviations from the mean of the group, indicating that the participant possibly had undiagnosed sensory neuropathy and was therefore inappropriate as a control.

The two groups consisted entirely of women. The bias toward female predominance is common in sub‐Saharan African HIV cohorts; further, studies do not report an influence of sex on HIV‐PN risk (Maritz et al., 2010 *,* 233 of 291 women; Vermaak, Dave, Levitt, & Heckmann, 2015 *,* 55 of 67 women; Borkum et al., 2017, 61 of 67 women).

The HIV‐infected group participants were all virally suppressed on ART (viral loads < 400 cps/ml) (Borkum et al., 2017) except one individual (≈4,000 cps/ml). In total, 15 of 20 had started ART prior to 2010 when the governmental‐sponsored ART program included stavudine. The current ART regimen comprised tenofovir, efavirenz, and emtricitabine in 14 of 20 (70%), which was introduced on average 7.5 years previously when the WHO recommended stavudine be discontinued. Other ART regimens included the following: 3 of 20 receiving zidovudine, efavirenz, lamivudine (9–10 years); 2 of 20 on second‐line ART, lopinavir/ritonavir, lamivudine, and zidovudine/efavirenz; and one person has been on stavudine, efavirenz, and lamivudine since initiating ART eight years ago.

Table 1 shows the participant characteristics. The HIV‐PN participants and controls were matched for age, foot dominance, and height. Matching for age is important since it is expected for age to have an effect on VPT. Weight and BMI were significantly different with the average control participant's body weight in the obese range (BMI > 30) which is common for this demographic (Malhotra et al., 2008). The difference in weight is likely a result of HIV infection and its treatment causing weight loss (Tang et al., 2002).

**Table 1:** Participant characteristics (mean and standard deviation in brackets)

	HIV‐PN	Controls	*p*‐value
Age (years)	41.6 (7.0)	40.1 (5.1)	0.449
Height (cm)	156.6 (6.1)	159.4 (5.3)	0.146
Weight (kg)	66.9 (13.8)	84.7 (23.0)	0.005
BMI (kg/m^2^)	27.1 (4.3)	33.2 (8.3)	0.006
Foot dominance (Right:Left)	19:1	16:3	0.342
ART duration (years)	8.6 (2.8)	‐	‐
BPNS‐total (0–11)	4.3 (1.1)	‐	‐
TNSr‐total (0–20)	4.9 (2.5)	‐	‐

Note

ART: antiretroviral therapy; BMI: body mass index; BPNS‐total: total score of Brief Peripheral Neuropathy Screening tool; TNSr‐total: total score of the reduced Total Neuropathy Screen.

In the HIV‐PN group, 18 of 20 had two or more neuropathic signs, and 2 had absent reflexes only. Only 2 cases experienced neuropathic symptoms. Table 2 shows the outcomes of the various assessments performed on the HIV‐PN group. In summary, the neuropathic signs in order of frequency included the following: 18 had altered/absent ankle reflexes (in 10, all deep tendon reflexes were altered/absent), 14 had reduced distal pinprick sensibility, 10 had altered vibration sensibility by TF, and 7 had altered toe proprioception. Of those with altered vibration sensibility, 50% had altered vibration at the toes only, and in the remainder, it extended more proximally.

**Table 2:** Frequencies of neuropathic signs for the HIV‐PN group

	Test score				
	0	1	2	3	4
BPNS‐TF	11	7	2	0	NA
BPNS‐DTR	NA	NA	2^a^	6	12
TNSr‐TF	10	5	2	2	1
TNSr‐DTR	2	6	2	7	3
Proprioception	13	7	0	NA	NA
TNSr‐pin Sensitivity	6	1	7	5	1

Notes

A score of zero indicates “normal” unless otherwise stated. Higher scores indicate more severe signs of neuropathy.

“NA” indicates that this score category does not apply to this test.

BPNS‐TF and BPNS‐DTR: Brief Peripheral Neuropathy Screening tool Tuning Fork and Deep Tendon Reflex evaluation. TNSr‐TF, TNSr‐DTR, and TNSr‐pin sensitivity: reduced Total Neuropathy Screen Tuning Fork score, Deep Tendon Reflexes score, and pin‐sensitivity score, respectively.

a A score of 2 for BPNS‐DTR indicates “normal.”

### 3.2 Comparison of VPT between HIV‐PN and control participants

**Figure 3:**
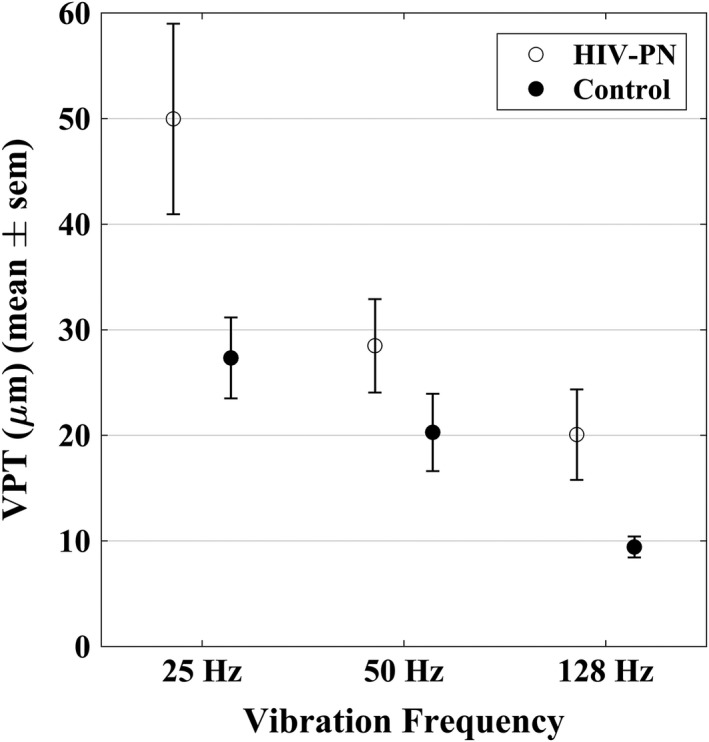
Vibration thresholds of HIV‐PN and control groups at three different Hz. Sensitivity was reduced in the HIV‐PN group at all frequencies (*p* = 0.018) compared to the controls

Figure 3 shows a comparison of the HIV‐PN group and the control group for VPT‐25, VPT‐50, and VPT‐128. HIV‐PN status significantly affected VPT overall (*p* = 0.018, *χ* ^2^ = 5.59, *df* = 1). Vibration Hz also significantly affected VPT (*p* < 0.001, *χ* ^2^ = 38.71, *df* = 2); that is, sensitivity increased with increases in vibration Hz. Although not statistically significant, an interaction effect between vibration Hz and HIV‐PN status on VPT suggests that HIV‐PN may affect VPT differently at different Hz compared to controls (*p* = 0.064, *χ* ^2^ = 5.51, *df *= 2). Post hoc analysis shows that HIV‐PN status had a more detrimental effect on VPT‐25 than VPT‐50 or VPT‐128.

Vibration perception threshold increased with age when controlling for vibration Hz and HIV‐PN status (*p* = 0.004, *χ* ^2 ^= 8.15, *df* = 1). However, age did not interact with vibration Hz (*p* = 0.60, *χ* ^2^ = 1.03, *df* = 2) or with HIV status (*p* = 0.123, *χ* ^2^ = 2.38, *df* = 1).

Vibration perception threshold did not correlate with participant height when controlling for vibration Hz and HIV‐PN status, nor did the interactions of height, and either vibration Hz or HIV‐PN status have a significant correlation with VPT (*p*'s > 0.57).

### 3.3 Comparison of VPT and clinical measures

There was no effect of ART duration on VPT (*p* = 0.53, *χ* ^2^ = 0.40, *df* = 1), but there was an interaction between ART duration and vibration frequency (*p* = 0.038, *χ* ^2^ = 6.56, *df* = 2). Post hoc analysis shows the patients who have been receiving ART for longer have improved sensitivity at 25 Hz. The sensitivity of other vibration Hz is largely unaffected by the number of years receiving ART.

**Figure 4:**
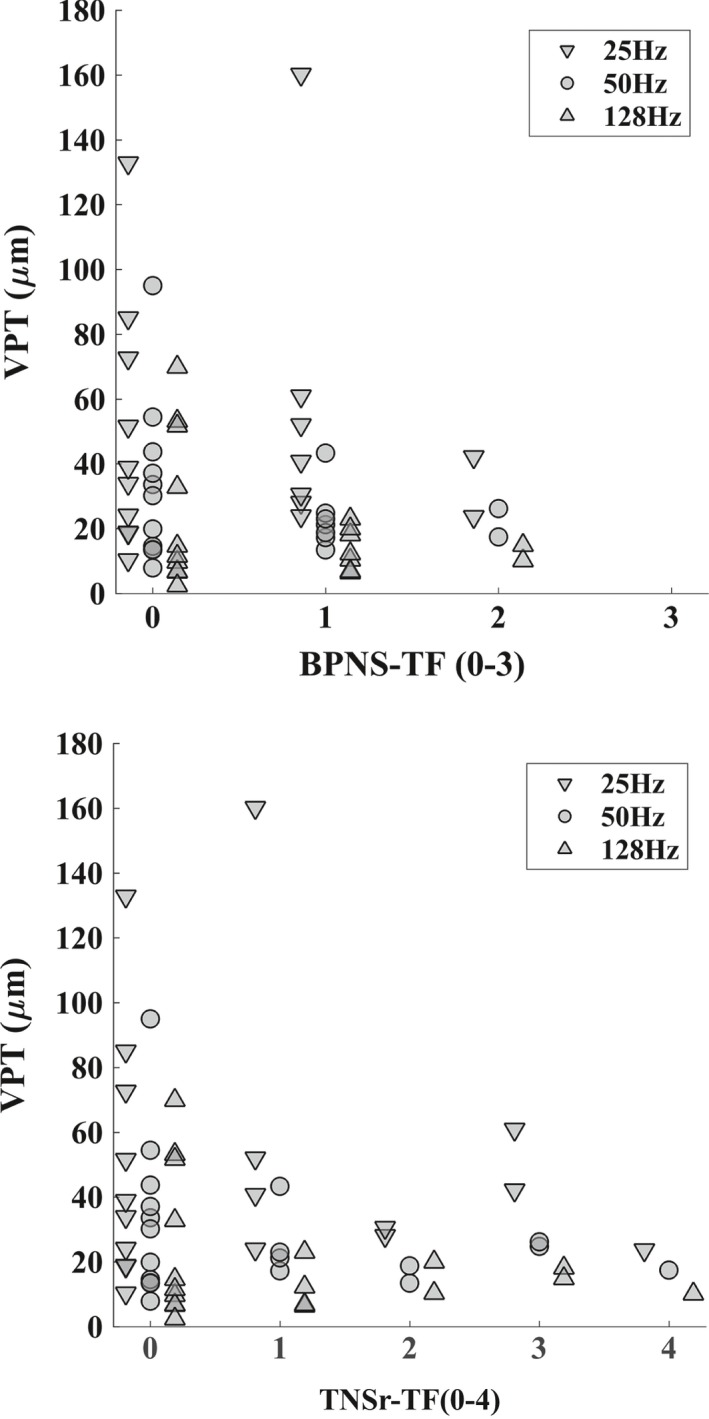
Plot of VPT at three different vibration Hz versus BPNS‐TF (top) and TNSr‐TF (bottom) for the HIV‐PN participants only. The data's scores are offset slightly for each vibration Hz to improve readability.

Figure 4 shows there was no relationship between vibrotactile sensitivity, as measured by VPT at different Hz, and the grading categories using the 128 Hz tuning fork at the toes (BPNS‐TF) or the length‐dependent vibration loss (TNSr‐TF). We found no significant correlation between VPT‐128 and BPNS‐TF, that is, at the toes (*p* = 0.76, ρ = −0.08) or TNSr‐TF, that is, distal–proximal extent (*p* = 0.90, ρ = −0.03). There was no significant effect of any of the clinical measures of large nerve fiber function as graded by BPNS‐TF, BPTNS‐DTR, TNSr‐TF, TNSr‐DTR, and proprioception on VPT or small fiber function as measured by pinprick sensibility (data table available in Supporting information Table S1, all *p*'s > 0.28). Neither was there any interaction with vibration Hz for large or small fiber function (*p*'s > 0.20).

## 4 DISCUSSION

This study presents an exploratory description of vibrotactile sensitivity in a subcohort of selected individuals with moderately severe predominantly asymptomatic HIV‐PN, using a robust psychophysics methodology, and compares the results to clinical screening tools commonly used in the study of HIV‐PN. The protocol used here, that differs from prior work, averages multiple measurements, uses a method of limits in both ascending and descending modes, is double‐blind, and reports the results in mechanical units (microns), making it more robust and potentially comparable across studies than currently available vibration testing. Previous studies using quantified sensory testing (QST) devices in HIV‐PN report their results in terms of mean Z‐scores based on their cohort controls scores (Phillips et al., 2014; Simpson, Kitch, & Evans, 2006). Therefore, we are not able to compare our results with previously reported HIV‐PN groups. Nevertheless, our control group was recruited using similar strategies to previous reports (Phillips et al., 2014).

The HIV‐PN group had reduced vibration sensitivity (raised VPT) compared to the control group. The sensitivity of both HIV‐PN and control groups increased with increase in vibration Hz in accordance with past research (Talbot, Darian‐Smith, Kornhuber, & Mountcastle, 1968). We would also expect to observe a decrease in sensitivity with increasing age (Mold et al., 2004) which was also shown to be the case for both HIV‐PN and control groups.

HIV‐PN is known to have length‐dependent properties (Centner et al., 2013, 2018); however, we found no correlation between height and VPT for either group. Subject heights in the HIV‐PN group were normally distributed with a standard deviation of only 6.1 cm; therefore, very few participants lay far from the mean to give strength to an analysis of height in this context. Although the lack of correlation should not be overinterpreted in this small sample, the absence of an association signal with height and HIV‐PN has been consistently absent in this population (Centner et al., 2018; Maritz et al., 2010).

An interesting result would be to find that HIV‐PN affects vibration sensitivity of different mechanoreceptor types differently. This could manifest when measuring VPT at different vibration Hz, as different mechanoreceptor populations have different vibration Hz responses. Indeed, Shy *et al.* note that different stimulation Hz hold different diagnostic value under varying circumstances (although they suggest testing above 128 Hz to more exclusively activate fast‐acting receptors) (Shy et al., 2003). We would observe this effect as an interaction between HIV‐PN status and vibration Hz when assessing VPT. Although this study was not statistically powered to find interaction effects which require significantly more statistical power, the interaction between HIV‐PN status and frequency, suggesting that HIV‐PN participants were more adversely affected at VPT‐25 than other test conditions (*p* = 0.0635), is interesting and will be discussed further below.

Previous work has shown that neuropathy symptoms and signs improve for some patients receiving ART over time and worsen for others (Centner et al., 2018). Although this cross‐sectional analysis did not find an overall effect of years on ART on VPT, post hoc analysis indicates that duration of ART was correlated with an improvement in 25 Hz VPT performance. Summarizing the results of the intergroup VPT analysis and the intragroup analysis of years on ART, we see HIV‐PN correlating with reduced sensitivity at all Hz, but particularly at 25 Hz, and that years on ART correlates with improved 25 Hz VPT performance. The observed interaction with 25 Hz in both cases is suggestive that the pathology preferentially affects slow acting mechanoreceptors and that treatment is partially effective in mitigating this.

All clinical measures failed to correlate with VPT. This lack of expected correlation can be explained through several mechanisms. Past results have shown that clinical measures of neuropathy have optimal diagnostic accuracy when combined with each other, including VPT (Pourhamidi, Dahlin, Englund, & Rolandsson, 2014; Shy et al., 2003). Excluding patients with severe painful neuropathy may have reduced the strength of the relationship between clinical measures and VPT, although there was no interaction with pinprick, which, like painful symptoms, subserves small fiber function. It is possible that the clinical measurements lack the resolution to demonstrate continuous relationships. For example, it has been shown that the relationship between the tuning fork vibration time and age only degrades about 5 s from the age of 20–60 on average (Alanazy, Alfurayh, Almweisheer, Aljafen, & Muayqil, 2018). It is therefore unreasonable to expect to see this relationship in a small cohort with a limited age range, and when tuning fork time is summarized into 5‐s blocks.

A limitation of this study is that the healthy controls did not undergo a neurological screen. This does not invalidate the results, but may have resulted in a smaller difference in VPT between the two groups than if potential control participants with undiagnosed neuropathy were excluded. Testing the control participants for HIV was not part of the protocol for ethical reasons. This omission is common for studies of this nature (e.g., Phillips et al., 2014).

Since SENS interventions have shown promise in improving VPT in the past with other populations that suffer from reduced VPT, it is conceivable that an intervention with SENS would be beneficial to this HIV‐PN population and should be explored as an option for therapy. The interaction between VPT‐25 and HIV‐PN status and between years on ART and VPT‐25 suggests that the response to vibration Hz may hold additional diagnostic value over using a tuning fork test only at 128 Hz.

## 5 CONCLUSION

To the best of our knowledge, this work presented the first quantitative, double‐blind measurement of VPT at different Hz in patients with HIV‐related sensory neuropathy reporting in mechanical units. Although this work is exploratory, the results indicate that patients with HIV‐PN have reduced vibration sensitivity at all tested vibration frequencies compared to age, height, and gender‐matched controls and that sensitivity increased with vibration frequency for both groups. The observation of an interaction with vibration Hz, particularly at 25 Hz, suggests that the pathology in subjects with HIV‐related neuropathy does not affect all mechanoreceptors similarly and requires further exploration.
