# Supplementary material for: Vibrotactile sensitivity of patients with HIV‐related sensory neuropathy: An exploratory study
Source: Brain Behav. 2018 Dec 18;9(1):e01184. doi: 10.1002/brb3.1184 (PMC6346661; doi:10.1002/brb3.1184)
Supplement: Supplementary file 1 [file BRB3-9-e01184-s001.pdf]

Supplementary Material

Table 1 Statistical outcomes for linear mixed effects models looking at the effect of various clinical bedside tests on VPT at all frequencies. DF = 1 for all main effects of the parameter on VPT, and DF = 2 for interaction with vibration frequency.

	**Main effect p (χ^2)**	**Interaction with frequency p (χ^2)**
**BPNS-TF**	0.41 (0.69)	0.79 (0.55)
**BPNS-DTR**	0.40 (0.71)	0.98 (0.05)
**BPNS-total**	0.50 (0.45)	0.96 (0.07)
**TNSr-TF**	0.34 (0.90)	0.99 (0.01)
**TNSr-DTR**	0.67 (0.19)	0.90 (0.21)
**TNSr-total**	0.99 (0.00)	0.80(0.44)
**PROP**	0.28 (1.18)	0.72 (0.66)
**TNSr-pin sensitivity**	0.46(0.55)	0.20(3.17)

BPNS-TF, BPNS-DTR and BPNS-total: brief peripheral neuropathy screening tool tuning fork evaluation, deep tendon reflex evaluation and total score respectively. TNSr-TF, TNSr-DTR, TNSr-total TNSr-pin sensitivity: reduced total neuropathy screen tuning fork score, deep tendon reflexes score, total score and pin sensitivity respectively. PROP: proprioception score. VPT: Vibration perception threshold. P, probability, χ^2, Chi squared value, DF, degrees of freedom.
